# Effect of alpha‐lipoic acid and myoinositol on endometrial inflammation in women with unexplained recurrent pregnancy loss

**DOI:** 10.1007/s00404-026-08365-8

**Published:** 2026-02-25

**Authors:** Chiara Tersigni, Maria Elisabeth Street, Roberta Castellani, Fiorella Di Nicuolo, Marianna Onori, Cecilia Catellani, Greta Barbaro, Carlo Ticconi, Nicoletta Di Simone

**Affiliations:** 1 Fondazione Policlinico Universitario A. Gemelli IRCCS, L.go A. Gemelli 8, 00168 Rome, Italy; 2 Department of Medicine and Surgery, University of Parma, Via Gramsci, 14, 43124 Parma, Italy; 3 Department of Mother and Child, University Hospital of Parma, Via Gramsci, 14, 43124 Parma, Italy; 4 Università Cattolica del Sacro Cuore, L.go F.Vito 1, 00168 Rome, Italy; 5 Department of Mother and Child, Azienda USL-IRCCS, di Reggio Emilia, Viale Risorgimento 80, 42123 Reggio Emilia, Italy; 6 Department of Surgical Sciences, Section of Gynecology and Obstetrics, University of Rome Tor Vergata, Via Cracovia 90, 00133 Rome, Italy; 7 Department of Biomedical Sciences, Humanitas University, Via Rita Levi Montalcini 4, Pieve Emanuele, 20072 Milan, Italy; 8 IRCCS Humanitas Research Hospital, Rozzano, 20089 Milan, Italy; 9 Istituto Scientifico Internazionale Paolo VI, Fondazione Policlinico Universitario A. Gemelli IRCCS, L.go A. Gemelli 8, 00168 Rome, Italy

**Keywords:** Alpha‐lipoic acid, Myoinositol, HMGB1, Inflammasome, Endometrium, Unexplained recurrent pregnancy loss

## Abstract

**Purpose:**

The aim of this study was to investigate the effect of oral administration of a combination of alpha- lipoic acid (ALA) and myo-inositol (Myo) on serum and endometrial inflammation of women with unexplained recurrent pregnancy loss (uRPL).

**Methods:**

Serum and endometrial levels of Nacht leucine-rich-repeat protein-3 (NLRP-3) and High Mobility Group Box 1 (HMGB-1) were analyzed by ELISA in women with unexplained Recurrent Pregnancy Loss (uRPL) (n = 31) and in control women who had had at least one uncomplicated pregnancy at term (n = 10) before and after daily oral administration of a commercial combination of ALA and Myo.

**Results:**

uRPL women had higher serum levels (p < 0.01) and endometrial content (p < 0.0001) of HMGB-1 than controls while no difference was observed regarding NALP3. Oral administration of ALA and Myo for three months significantly decreased pre-treatment serum levels of NLRP-3 (p < 0.0001) and HMGB-1 (p < 0.0001), as well as endometrial content of NLRP-3 (p < 0.001) and HMGB-1 (p < 0.0001) in uRPL women, similar to those found in controls.

**Conclusion:**

Women with uRPL have increased systemic and endometrial inflammation than controls. Oral administration of ALA and Myo could modulate serum and endometrial levels of NLRP-3 and HMGB-1 in uRPL women.

## What does this study adds to the clinical work?

**Table:** 

Women with unexplained recurrent pregnancy loss (uRPL) have increased systemic and endometrial inflammation compared to health women with uncomplicated pregnancy. Oral administration of ALA and Myo can reduce systemic and endometrial inflammation, by decreasing the levels of NLRP-3 and HMGB-1 in uRPL women.	

## Introduction

Recurrent Pregnancy Loss (RPL) is a clinical condition affecting up to 2% of couples trying to conceive [1]. According to ESHRE, it is defined as the occurrence of two or more miscarriages before 24 gestational weeks [2]. Main risk factors for RPL are maternal age and number of previous miscarriages [3]. A complete diagnostic work up should be offered to RPL couples to identify any treatable clinical condition predisposing to miscarriage. ESHRE guidelines recommend investigating female anatomical, autoimmune, genetic, hormonal and metabolic factors as well as male sperm anomalies [2]. Unfortunately, even after a complete work up in more than 50% of couples no causal factor for RPL can be found, defining the condition of unexplained RPL (uRPL) [4]. Growing evidence suggests a central role of the immune system in the pathogenesis of uRPL. Indeed, pregnancy is a special inflammatory and immunological condition, requiring both a systemic [5] and a uterine immunological adaptation [6] by the mother. It has become clear that the endometrium of uRPL women can have several immune dysfunctions [7] including an abnormal immune cell composition and cytokine imbalance [8, 9]. In particular, endometrial levels of interferon-γ (IFN-γ), interleukin-1β (IL-1β) and IL-18 have been shown to be higher in uRPL women than in women with successful pregnancy outcome [10–12]. Consistently, the endometrium of RPL women expresses increased levels of Nacht leucine‐rich‐repeat protein‐3 (NLRP‐3) inflammasome [13, 14], a multiprotein structure belonging to the innate immunity. NLRP-3 has a cascading activation mechanism, culminating with the release of mainly IL-1 and IL-18, molecules with a strong chemotactic and proliferative effect, promoting inflammation and coordinating innate and adaptive immune responses [15]. NLRP-3 inflammasome can be activated by an extremely wide range of triggers, commonly classified as Pathogen-Associated Patterns (PAMPs) or Damage-Associated Molecular Patterns (DAMPs). The latter are released by damaged or dying cells and are recognized by PRRs (Pattern Recognition Receptors), such as Toll-like receptors (TLRs) and cytoplasmic Nod-like receptors (NLRs) [16].

Among DAMPs, High mobility Group Box 1 (HMGB-1) is a DNA-binding protein, normally located inside the cellular nucleus, where it is involved in the process of chromatin remodeling. HMBG1 can be released either actively from immunologically activated immune cells under distress conditions or passively from damaged cells, during necrosis or apoptosis, leading to HMGB-1 spreading outside the cell [17, 18]. HMGB-1 is a strong inducer of systemic and local inflammatory response, activating TLRs both in immune and in non-immune cells, like endothelial cells, epithelial cells and fibroblasts [19]. Interestingly, secretion of HMGB-1 from macrophages is associated with inflammasome NLRP-3 activity that allows HMGB-1 translocation from the nucleus to cytoplasm [20]. Thus, HMGB-1 release is tightly associated with inflammasome activation and can be considered a marker of both tissue and systemic inflammation. Consistently with the higher endometrial activation of NLRP-3 in RPL patients, increased serum and endometrial concentrations of HMGB-1 have been shown in RPL women [21, 22].

Serum and ovarian levels of HMGB-1 are also increased in the polycystic ovarian syndrome (PCOS), another chronic inflammatory condition [23]. Previous reports have shown that oral administration of α-lipoic acid (ALA) and Myo-inositol (Myo) can reduce both endometrial inflammasome NLRP-3 in RPL women [24] and serum levels of HMGB1 in PCOS women [23, 25]. ALA is a molecule widely spread in foods and normally found in mitochondria, with antioxidant properties, since it is able to counter Reagent Oxygen Species (ROS) formation and can also enhance the activity of other endogenous antioxidants (such as vitamin C, vitamin E and glutathione) [26]. Furthermore, ALA exerts a direct anti-inflammatory action, by modulating the NF-kB signaling pathway, preventing it from binding to DNA and inducing transcription of pro-inflammatory proteins [27, 28].

Myo has an insulin-sensitizing effect on endometrial cells [29] and it can indirectly reduce endometrial oxidative stress and inflammation by reducing insulin resistance and hyperglycemic state associated with ROS generation [30, 31].

Since modulation of endometrial inflammation is supposed to improve uterine environment and might potentially increase pregnancy rate and live births in uRPL couples, the main aims of this study were: a) to assess serum and endometrial levels of HMGB-1 and NLRP-3 in a cohort of uRPL women compared to women with two or more previous uncomplicated pregnancies; b) to study the effect of oral administration of a combination of ALA and Myo for three months on serum and endometrial HMGB-1 and NLRP-3 levels in a cohort of uRPL women.

## Materials and methods

### Study population

This study was conducted at the Department of Woman and Child Health of the Fondazione Policlinico A. Gemelli IRCCS of Rome, Italy. The study was approved by the Ethics Committee of the Fondazione Policlinico A. Gemelli IRCCS of Rome (ID: 1355 date of approval 30/01/2017) and conducted following the principles of the Declaration of Helsinki. Women with uRPL, referred to our Recurrent Pregnancy Loss Unit from January 2019 to March 2021, were recruited in this study. They underwent a standardized diagnostic workup already reported in detail [32] and were diagnosed to have uRPL when no causes/risk factors could be identified. Control women were patients who underwent hysteroscopy for ultrasound suspicion of endometrial polyp, unconfirmed by hysteroscopy. They had at least one uncomplicated pregnancy at term without any pregnancy loss. Written informed consent was collected from all patients enrolled in the study. Clinical data from all recruited women were collected, anonymized and analysed.

### Treatment

Based on previous evidence of the anti-inflammatory effects of oral administration Ala and Myo for three months on the endometrium of RPL women [24], all the uRPL women recruited in this study received twice a day oral administration of one commercial tablet of Sinopol ®, containing ALA (400 mg) plus Myo (500 mg) for three months. They were followed up through a monthly telephone call to assess the adherence to the treatment. At the end of the treatment, they were interviewed to evaluate the continuous adherence to the treatment. The control group women received no drugs or other nutraceutical agents.

### Outcome of the successive pregnancy in women with uRPL

A 48 months follow-up was conducted to evaluate the pregnancy rate and live birth rate in the subsequent pregnancy in all women enrolled in the study.

### Serum collection

Venous blood samples (3 ml) were collected from uRPL and control women at recruitment, and after 3 months of treatment with ALA plus Myo (uRPL women only), through antecubital venipuncture. Each sample was centrifuged at 1200 g for 10 min at 20 °C to remove the cellular component and was divided in 500 μl aliquots and frozen at −80 °C until further use.

### Endometrial sampling

Both uRPL and controls women underwent hysteroscopic biopsy during the putative window of implantation (days 22th-24th of the menstrual cycle also checked by the urinary LH surge—LH surge + 7–9 days). RPL women underwent a second hysteroscopic biopsy—carried out at the same menstrual period determined as above—after three months of oral administration of ALA plus Myo. Endometrial biopsies were performed using a 3‐mm Novak curette. Each sample was divided into two aliquots, for the conventional histology and for the study purpose. Study specimens were washed immediately in normal saline solution. Lysates were obtained using the modified RIPA lysis buffer (Thermo Fisher, Waltham, Massachusetts, USA) containing Na3VO4 as phosphatase inhibitor, PMSF and leupeptin as protease inhibitors. The total protein content was determined by Bradford assay, and it was expressed in micrograms per milligrams (μg/mg) of endometrium. Samples were stored at −80 °C until further use.

### Elisa assay

NLRP-3 and HMGB-1 were measured in serum and in endometrial lysates by using commercial ELISA kits (Abcam, Cambridge, UK and TECAN, Mannendorf, Switzerland, respectively). One hundred microliters of samples were used for each well. The experiments were conducted in duplicate, according to the manufacturer’s recommended protocols. Intra-assay and inter-assay CVs were 5.4% and 8.2%, respectively; detection limit was 0.15 ng/ml. Concentrations were normalized per mg of total protein content as specified above.

### Statistical analysis

Data are expressed as mean ± SD or median with interquartile range (IQR), according to their distribution previously investigated by using the Shapiro–Wilk test. Clinical characteristics of the population were analyzed by the student’s t test and the Chi-square test. Normally distributed variables were analyzed by the Student’s t test. Not normally distributed variables were analyzed by the Kruskal–Wallis and the Mann–Whitney U tests, as appropriate. Paired data were analyzed using the Wilcoxon matched-pairs test, appropriate for non-parametric data. Dunn test was also used as post hoc test when necessary. Statistical analysis was performed with Prism software version 10.0. For all analyses, p < 0.05 was considered significant.

## Results

### Characteristics of the study population

**Fig. 1:**
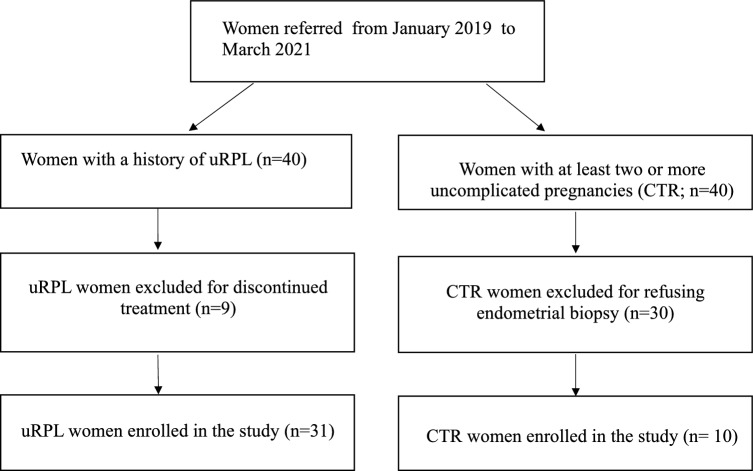
Flow chart summarizing inclusion, exclusion, and drop out of participants of this study

In this study, 31 women with a history of uRPL and 10 women with at least one previous uncomplicated pregnancy at term without any miscarriage were recruited. Details about selection of patients are shown in Fig. 1. The clinical characteristics of the study population are shown in Table 1.

**Table 1:** Clinical characteristics of uRPL women and controls (CTR) women enrolled in this study

	uRPL (n = 31)	CTR (n = 10)	P
Age (yr)	36.3 ± 4.9	36.1 ± 6.6	0.6
BMI (Kg/m2)	22.2 ± 3.5	21.5 ± 2.9	0.5
Total number of pregnancies (n)	3.0 (2–5)	2.0 (1–2)	< 0.0001
Live births (n)	0 (0%)	18 (100%)	< 0.0001
Pregnancy losses (n)	98 (100%)	0 (0%)	< 0.0001
Pregnancy losses per woman (n)	3 (2–5)	0	< 0.0001
Number of live births per woman (n)	0	2 (1–2)	< 0.0001

Results are expressed as mean ± SD, median and interquartile range (IQR) or percentages. The analysis was performed by the Mann–Whitney U test and the Chi-squared test, as appropriate

*CTR* women with previous uncomplicated pregnancies, *BMI* body mass index, *uRPL* unexplained recurrent pregnancy loss

### Serum levels of NLRP-3 and HMGB-1 in study women

**Fig. 2:**
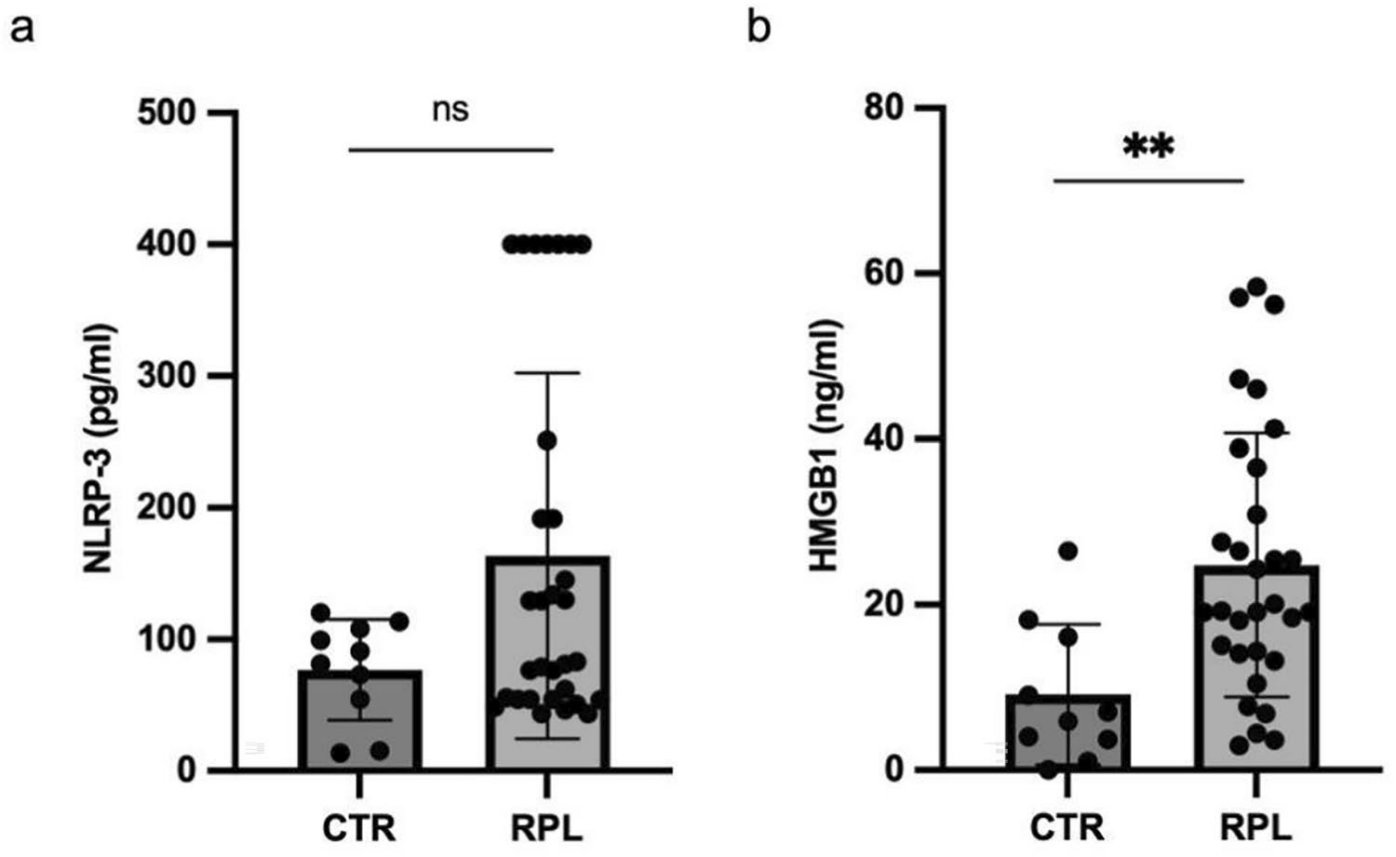
Scatter plot representing serum levels of NLRP-3 (**a**) and HMGB-1 (**b**) in uRPL women (n = 31) compared to CTR (n = 10). Results are expressed as median ± interquartile range (IQR). *RPL* unexplained recurrent pregnancy loss, *CTR* controls, *ns* not significant; **p < 0.01

No significant differences in serum levels of NLRP-3 were observed between uRPL and control women (Fig. 2a). Women with uRPL had significantly higher serum levels of HMGB-1 than control women (Fig. 2b).

### uRPL women had higher levels of HMGB-1 in endometrial lysates

**Fig. 3:**
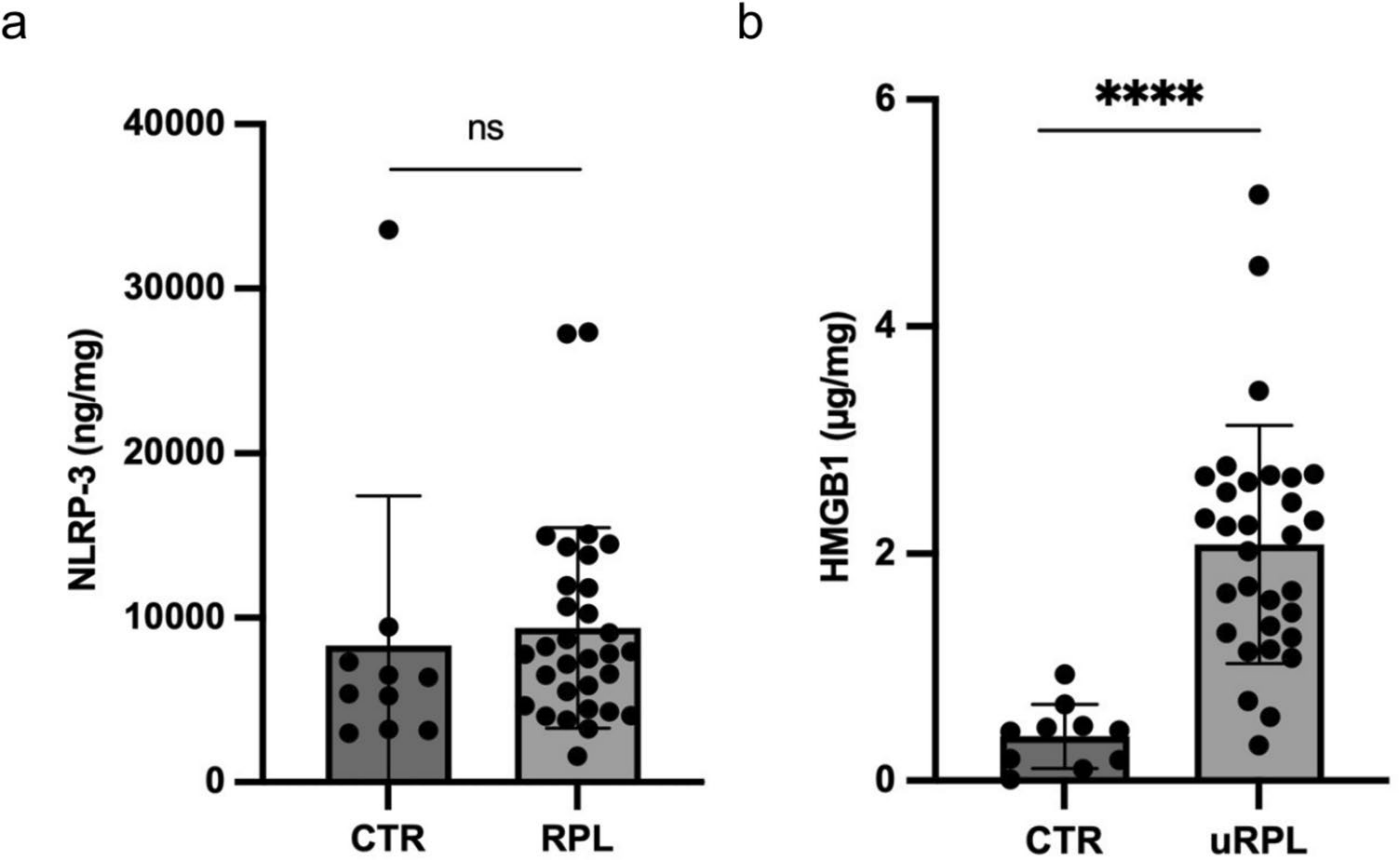
Scatter plot representing normalized NLRP-3 (**a**) and HMGB-1 (**b**) concentrations in endometrial lysates in uRPL women (n = 31) compared to CTR (n = 10). Results are expressed as median ± interquartile range (IQR). *RPL* unexplained recurrent pregnancy loss, *CTR* controls, *ns* not significant; ****p < 0.0001

No significant differences in endometrial levels of NLRP-3 were detected between uRPL and control women (Fig. 3a). An increased concentration of HMGB-1 was observed in uRPL women compared to the control ones (Fig. 3b).

### ALA plus Myo reduced serum levels of NLRP-3 and HMGB-1 in RPL women

**Fig. 4:**
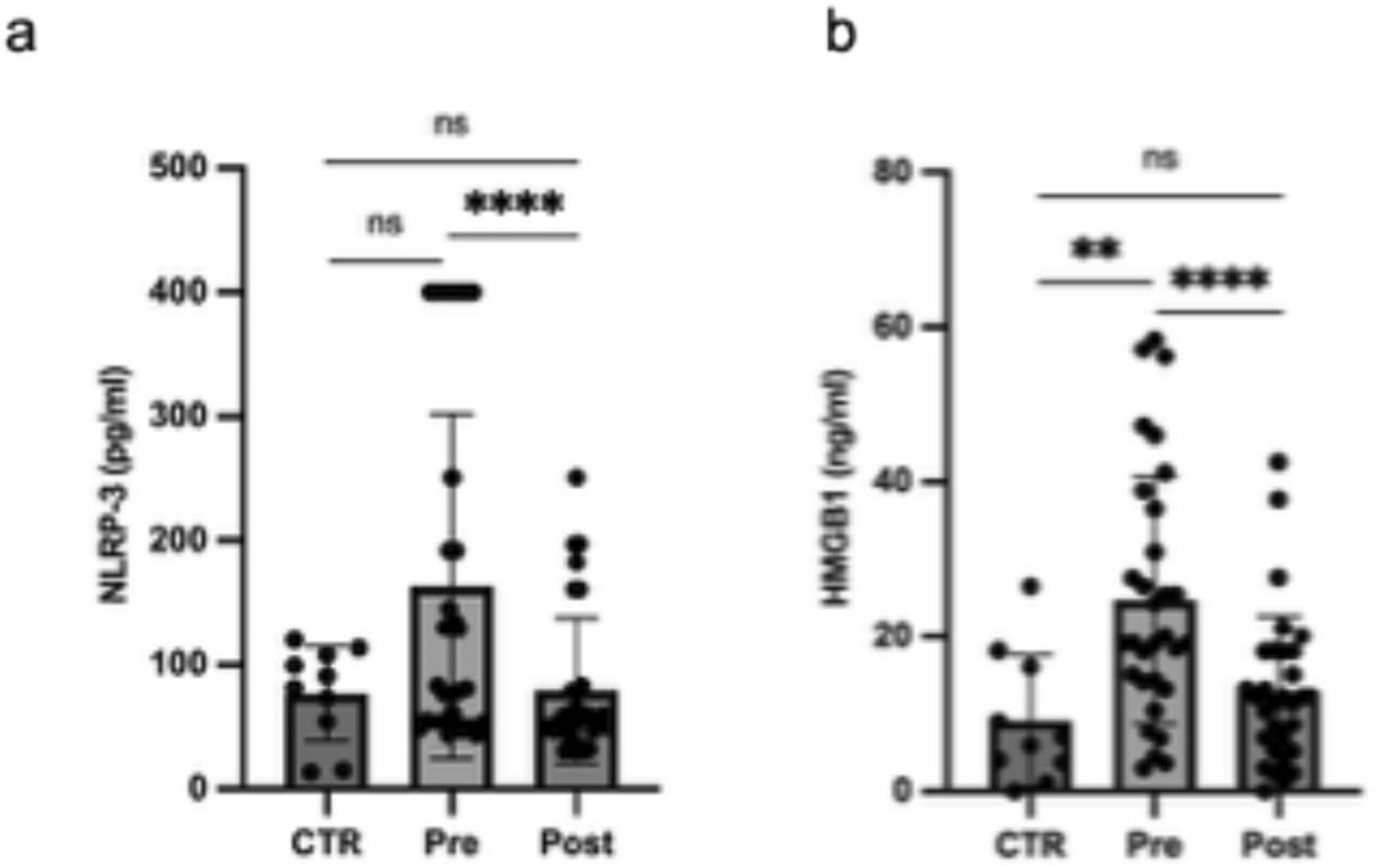
Scatter plot representing serum levels of NLRP-3 (**a**) and HMGB-1 (**b**) in uRPL women, pre- and post-administration of ALA plus Myo, compared with untreated controls. Results are expressed as median ± interquartile range (IQR). *Pre* unexplained recurrent pregnancy loss pre-treatment, *Post* unexplained recurrent pregnancy loss post-treatment, *CTR* controls, *ns* not significant, *p < 0.05; **p < 0.01, **** p < 0.0001

Decreased serum levels of NLRP-3 and HMGB-1 were observed in uRPL women after 3 months of oral administration ALA plus Myo compared to pre-treatment levels (Fig. 4).

### ALA plus Myo reduced NLRP-3 and HMGB-1 in endometrial lysates in RPL women

**Fig. 5:**
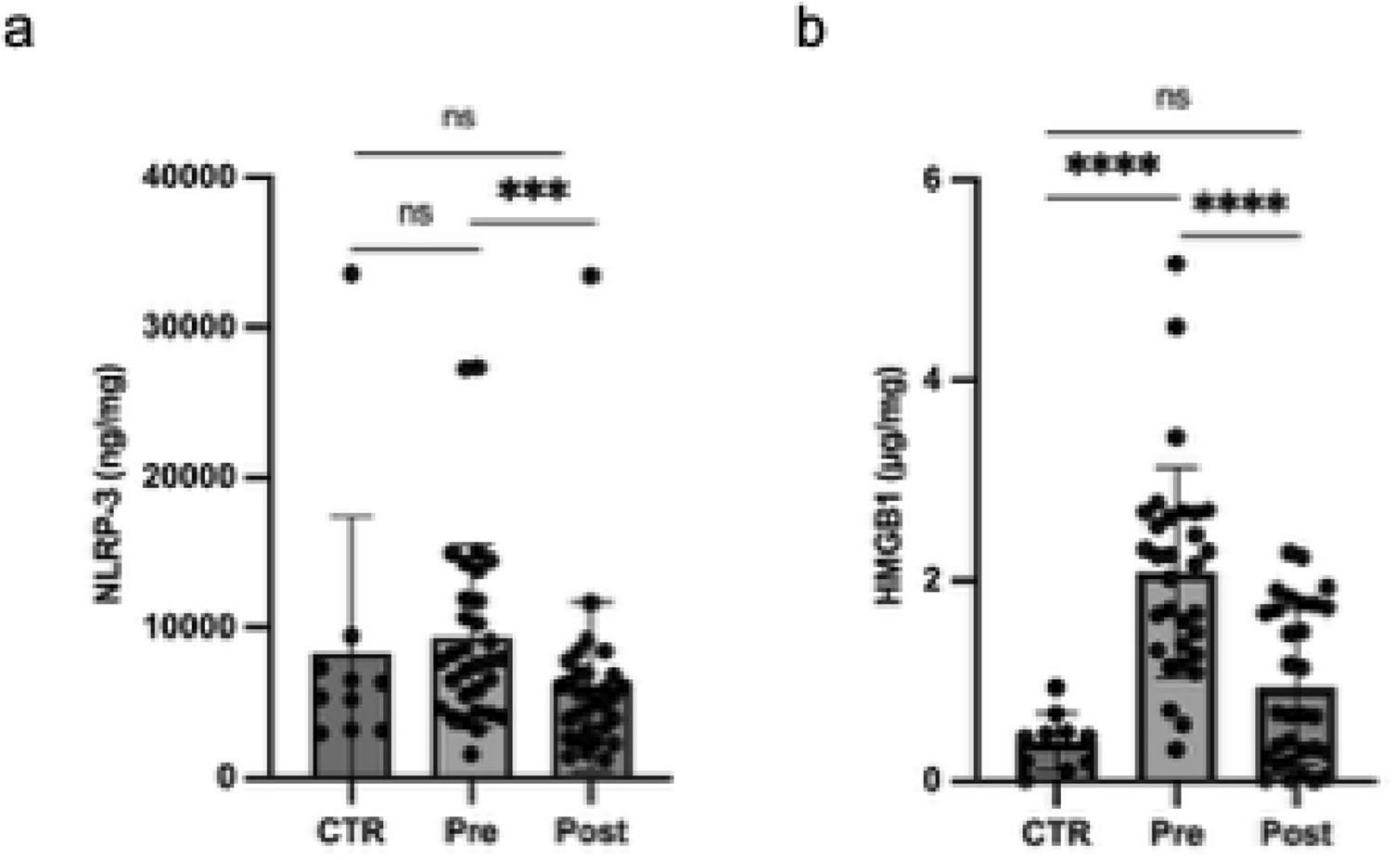
Scatter plot representing normalized NLRP-3 (**a**) and HMGB-1 (**b**) in endometrial lysates in RPL women, pre- and post- administration of ALA plus MYO, and in untreated controls. Results are expressed as median ± interquartile range (IQR). *Pre* unexplained recurrent pregnancy loss pre-treatment; *Post* unexplained recurrent pregnancy loss post-treatment; *CTR* controls; *ns* not significant; ***p < 0.001; ****p < 0.0001

Lower endometrial levels of NLRP-3 and HMGB-1 were observed in uRPL women after 3 months of oral administration with ALA plus Myo compared to pre-treatment levels (Fig. 5).

### Live birth rate of RPL women administered with ALA plus Myo

Higher live birth rate in the subsequent pregnancy was observed in RPL women after three months oral administration of ALA plus Myo (Table 2).

**Table 2:** Obstetric outcomes of uRPL women after administration of ALA plus Myo

	uRPL pre (n = 31)	uRPL post (n = 31)	P
Live births (n)	0 (0%)	39 (64.5%)	< 0.0001
Pregnancy losses (n)	98 (100%)	11 (35.5%)	< 0.0001
Pregnancy losses per woman (n)	3.2 ± 0.9	1.0 ± 0.0	< 0.0001
Number of live births per woman (n)	0.0 ± 0.0	1.7 ± 0.5	< 0.0001

Results are expressed as median and interquartile range (IQR) or percentage, according to variables, and were considered statistically significant for p < 0.05. The analysis was performed by the Mann–Whitney U test and the Chi-squared test, as appropriate

*uRPL* unexplained recurrent pregnancy loss, *Pre* unexplained recurrent pregnancy loss pre-treatment, *Post* unexplained recurrent pregnancy loss post-treatment

## Discussion

In this study we have shown for the first time that the administration of ALA plus Myo reduces endometrial and systemic levels of HMGB-1 in women with uRPL. Furthermore, we confirmed previous reports showing higher endometrial levels of HMGB-1 in women with uRPL [22]. Finally, we confirmed the inhibitory effect of ALA plus Myo on endometrial inflammasome NLRP-3 in uRPL women, consistently with a previous work from this research group [24].

It can be speculated that the possible mechanisms of action linking NLRP-3 and HMGB-1 might be related with the ability of HMGB-1 to bind TLRs and activate the NLRP-3 cascade, via NF-κB intracellular signaling. Furthermore, once NLRP-3 is assembled, its activation cascade can lead in turn to HMGB-1 translocation from the cell nucleus to the membrane [33], determining positive feedback and an auto-activation cascade.

Intriguingly, in both humans and rats, significantly lower endometrial levels of HMGB-1 were observed in the receptive phase of the menstrual cycle compared to the pre-receptive phase, together with a significant reduction of endometrial HMGB-1 on the day of implantation in pregnant rats. Furthermore, administration of HMGB-1 to pregnant-rats induces pregnancy failure [34]. Thus, low levels of endometrial HMGB-1 seem to be crucial for a successful embryo implantation and a good pregnancy outcome.

Supporting this hypothesis, in a mouse model of RPL, HMGB-1, NLRP-3, Caspase-1, TLR2 and TLR4 were demonstrated to be highly expressed and NF-κB signaling pathway was activated in the decidua tissue [35].

The upregulation of HMGB-1 at the maternal–fetal interface (villi and decidua) of uRPL women has been proposed to be related to macrophage cells infiltration that are able to release HMGB-1 by activating NF-κB via TLRs [21].

Since ALA can inhibit NF-κB signaling pathway activation [36] and NF-κB is a key intracellular factor involved in the transcription of NLRP-3, IL-1β and IL-18 after TLRs stimulation [37], we can speculate that ALA could reduce both HMGB-1 and NLRP-3 expression through inhibition of NF-κB signaling. This hypothesis has not been tested in this study and further research is required to investigate a possible involvement of NF-κB in the ALA-induced reduction of serum and endometrial levels of NLRP-3 and HMGB-1. Another possible mechanism to be considered is that Myo might modulate endometrial inflammation by reducing insulin resistance. Indeed, hyperglycemic state is associated with ROS generation, potentially leading to cell autophagy and DAMPs release [30, 38]. More research is required to disentangle the molecular mechanisms of action of ALA and Myo on endometrial inflammation in larger cohorts.

The main limitations of this study are: a) the small size of the control group, due to the difficulty to get endometrial samples from healthy fertile women; b) the absence of a placebo group; c) the lack of a common diet regimen in cases and controls.

## Conclusions

Oral administration of ALA and Myo to uRPL women seems to decrease serum and endometrial levels of NLRP-3 and HMGB-1 compared to pre-treatment levels, modulating systemic and endometrial inflammation to levels like controls. Further research is needed to investigate the molecular mechanisms of action of ALA and Myo on endometrial and systemic expression of NLRP-3 and HMGB-1 in RPL, and to verify whether this molecular complex of innate immunity might be a new therapeutic target to manage the complex and heterogeneous syndrome of RPL.

## Data Availability

No datasets were generated or analysed during the current study.
